# Effect of Space Flight Factor on Dormant Stages in Aquatic Organisms: A Review of International Space Station and Terrestrial Experiments

**DOI:** 10.3390/life12010047

**Published:** 2021-12-29

**Authors:** Victor R. Alekseev, Jiang-Shiou Hwang, Margarita A. Levinskikh

**Affiliations:** 1 Zoological Institute RAS, 199034 St. Petersburg, Russia; 2 Institute of Marine Biology, National Taiwan Ocean University, Keelung 20224, Taiwan; 3 Center of Excellence for Ocean Engineering, National Taiwan Ocean University, Keelung 20224, Taiwan; 4 Center of Excellence for the Oceans, National Taiwan Ocean University, Keelung 20224, Taiwan; 5 Institute of Biomedical Problems of the Russian Academy of Sciences, 123007 Moscow, Russia; ritalev@imbp.ru

**Keywords:** crustacean dormant stages, open space conditions, diapause, space flight factors, ISS, interplanetary quarantine, astrobiology, hydrobiology, microgravity, cosmic radiation, ultraviolet light, magnetic field, temperature effects, search for extraterrestrial life forms

## Abstract

This work is a review of the experiments carried out in the Russian segment of the ISS (inside and outside) from 2005 to 2016 on the effect of the space flight factor on the resting stages of organisms. In outer space, ultraviolet, a wide range of high and low temperatures, cosmic radiation, altered gravity, modified electromagnetic field, vacuum, factors of technical origin, ultrasound, microwave radiation, etc. and their combination determine the damaging effect on living organisms. At the same time, biological dormancy, known in a wide range of bacteria, fungi, animals and plants, allows them to maintain the viability of their dormant stages in extreme conditions for a long time, which possibly allows them to survive during space flight. From 2005 to 2016, the resting stages (propagules) of micro- and multicellular organisms were tested on the ISS to assess their ability to survive after prolonged exposure to the conditions of open space and space flight. Among the more than 40 species studied, about a third were dormant stages of aquatic organisms (eggs of cyprinodont fish, daphnia embryos, resting eggs of fairy shrimps, tadpole shrimps, copepods and ostracods, diapausing larvae of dipterans, as well as resting cysts of algae). The experiments were carried out within the framework of four research programs: (1) inside the ISS with a limited set of investigated species (Akvarium program); (2) outside the station in outer space without exposure to ultraviolet radiation (Biorisk program); (3) under modified space conditions simulating the surface of Mars (Expose program); and (4) in an Earth-based laboratory where single-factor experiments were carried out with neutron radiation, modified magnetic field, microwave radiation and ultrasound. Fundamentally new data were obtained on the stability of the resting stages of aquatic organisms exposed to the factors of the space environment, which modified the idea of the possibility of bringing Earth life forms to other planets with spacecraft and astronauts. It also can be used for creating an extraterrestrial artificial ecosystem and searching for extraterrestrial life.

## 1. Introduction

Investigations to advance the use of animal and plant anabiosis, e.g., cryptobiosis and some other forms of dormancy, in space exploration highlight five notable programs on exobiology. Biomedical support of humans in the absence of the factors important to the sustenance and development of every living thing is one of the indisputable aspects of space exploration [1].

The development of life support systems (LSS), including systems incorporating the biological cycle, has been pursued since the initial space flights of cosmonauts [2,3,4,5,6].

The implementation of the central ecological life support systems (CELSS) for space crews requires prior all-around tests and studies in order to: determine the biological impacts of the space flight factors on the life of individual organisms as well as communities (populations and biocenoses); develop technologies for cultivating highly productive populations of autotrophs and heterotrophs in a zero-gravity environment; design hardware to sustain the vital functions of autotrophs and heterotrophs as members of the space crew CELSS; search for methods to preserve the gene pool aboard the space vehicle and on the planetary outposts; and optimize the CELSS with consideration for microgravity and constant radiation exposure. The phenomenon of protracted biological resting can be viewed as the alternative to transportation of the active ecosystem [7].

Persisting forms of life may be the cause for incidental colonization of planets by terrestrial organisms and vice versa. This illustrates the problem of biological survival in hostile environments on and beyond Earth. The quarantine measures to be developed should establish a barrier to any illicit penetration of dormant life into the environment of Earth or another planet.

To discover life in extraterrestrial ecosystems utterly different than our own, search technologies may be required based on an intimate knowledge of cryptobiosis. A comparison of the survival time of dormant organisms during cryptobiosis shows that cysts of unicellular organisms (equally animals and plants, e.g., protozoa and microalgae, respectively) that were first dried and then frozen in ice displayed an amazing ability to revive after as long as many thousand years of dormancy [8].

This long maintenance of life provides evidence of the possibility of an interplanetary transfer of cryptic forms of life on meteorites, dust, and the ice particulates of comets.

The work is a review of experiments carried out in the Russian segment of the ISS from 2005 to 2016, including several space experiments (Akvarium, Biorisk, Expose-R, Expose-R2, Phobos-Grunt), which involved the resting stages of a large number of organisms from bacteria to fish (Table 1).

The combination of the various conditions in these experiments, from the conditions on board a spacecraft to the conditions in open space, made it possible to assess the impact of various elements of the so-called factor of space flight (SFF) (Table 2).

This article, which is a review of the publication of a large number of participants in these experiments, examines the comparative effects of the elements, studied both in space and in terrestrial conditions.

To unify the assessment of the impact of the factors, the cladoceran species *Daphnia magna*, a very common water test organism in terrestrial experiments, was used.

The results obtained at resting stages of other aquatic organisms, such as brine shrimp, streptocephalus and ostracods, generally did not contradict the results established for *Daphnia magna* and, in many cases, supported them [19,20,21].

A general program for the study of biological dormancy was outlined in the beginning of the century [1,22]; it included several directions:

- The study of the possibility of uncontrolled transfer of living organisms to other planets (interplanetary quarantine);
- The maintenance of microbiological safety on space ships;
- The study of the possibility of searching for extraterrestrial life on other planets, primarily on Mars;
- The creation and transportation of biological systems for recycling oxygen, water and food in artificial extraterrestrial conditions.

To solve most of these issues, it turned out to be necessary to assess the impact of space flight.

The important elements of the SFF on an open space that negatively affect living organisms are:

Solar radiation, a large temperature difference from +100 to −100 °C, corpuscular cosmic radiation and vacuum; on board: micro gravitation, modified magnetic field, neutron radiation as a result of the passage of high-energy cosmic particles through the ship, technical noise, ultrasound and microwave radiation. Acting separately as well as together (synergistic effect), these elements of the SFF should have an impact, not only on active organisms, but also on the viability of their dormant stages that are well prepared for survival in the harsh conditions of the Earth.

In this review, the authors give an outline of each program and quantitatively evaluate the role of each of the elements of the space flight factor in order to expand the understanding of the stability of life in the adverse conditions of space and to use this data for the exploration of other planets and the search for extraterrestrial life.

It should be said that the highly protective properties in the dormant stages of organisms against the effects of negative elements of the SFF are fundamentally different from the mechanisms and possibilities for overcoming these negative effects in the active stages of organisms, both aquatic and terrestrial. The increased viability of the diapausing stages of invertebrates and fish is based on a reduced level of the general metabolism of the organism and its related functions such as respiration, feeding, movement, etc., which are reduced to zero in the stages most deeply immersed in biological dormancy. This determined the possibility of long-term (up to 2 years in the conducted experiments) maintenance of the viability of these stages in the atmosphere-deprived Biorisk and Expose-R modules on the outside of the ISS. The second important adaptation of these stages, which determined their increased resistance to significant temperature fluctuations outside the ISS as well as to the damaging effects of neutron radiation and the high-energy part of the solar radiation spectrum, is their almost complete dehydration and replacement of water in cells with protective antifreeze substances (trehalose, etc.). Finally, most of the organisms used in these experiments were embryos that stopped developing at the early stages of development (usually gastrula); these non-specialized cells have an increased resistance to negative influences in comparison with tissue cells, and the embryos themselves are enclosed in multilayer membranes that increase their protection from external mechanical, chemical and energy (ultraviolet) influences. Previous studies of the effects of the space flight factor were carried out on organisms of a wide evolutionary range from bacteria to humans but always in an active state (e.g., [23,24,25,26]). The level of adaptive capabilities of active organisms is not comparable with the resting stages, and the mechanisms of adaptation to the SFF are radically different for them. This determined the necessity of their separate study and consideration in the form of independent scientific directions [1,27].

## 2. Experiments Inside the Spacecraft in ISS Orbit

**Figure 1:**
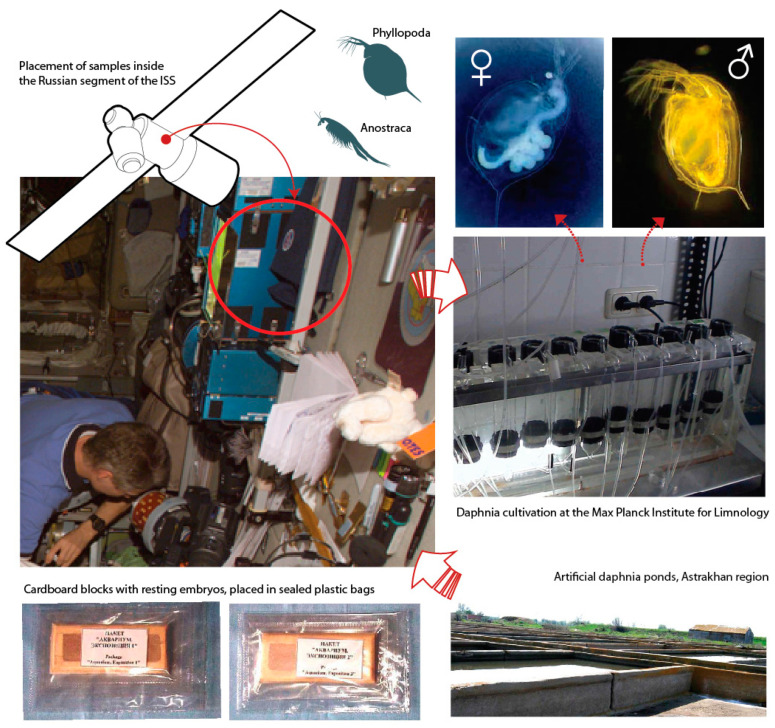
Akvarium program. Shows: experimental organisms; the artificial pond for growing daphnia, where the material was taken from; cardboard blocks with resting embryos, placed in sealed plastic bags; the sample holder is located next to the “Plants” block; cultivation of daphnia at the Max Planck Institute of Limnology (Ploen, Germany).

**Figure 2:**
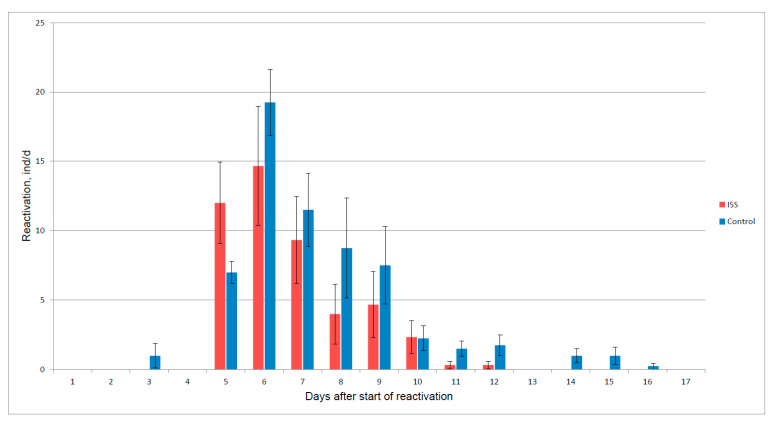
Reactivation of *Daphnia magna* in control and after flight. Bars illustrate the mean and standard error, *p* < 0.05.

The experiments of the Akvarium program (Figure 1) involved dry resting stages of two crustacean species. They were uniformly glued on special plates in one layer and placed in hermetically sealed plastic bags. On Earth, the control samples were stored in stable temperature conditions of two types: (1) in the dark at +4 °C and at room temperature (from +20 to +22 °C) and (2) in permanent light at room temperature (from +20 to +22 °C). The first option is considered optimal for storage, while the second repeated the temperature and light conditions on the ISS. After exposure on the ISS for 1 and 8 months, the experimental samples and control were delivered to the laboratory of the Max Planck Institute of Limnology, Ploen, Germany, where, under strictly controlled conditions, the following parameters were determined: percentage of reactivation, resistance to fungal diseases, dry weight of newborns, life cycle parameters (duration; maturation; number of eggs in the first, second and third clutches; as well as length and dry weight of the female after laying the first clutch of eggs). In the second generation, the sex ratio and the size and number of newborns in the offspring exposed on the ISS, as well as the control samples, were assessed [28,29]. A comparison of the experimental and control organisms that emerged from the resting eggs revealed a decrease in a number of indicators in the individuals exposed in space. Differences in the percentage of reactivation, the timing of reaching maturity, and the number of eggs in the first clutch were statistically significant. In the second generation, the individuals exposed on the ISS reacted to this with a sharp increase in the number of males (30–78% of offspring in third clutch), which was not observed in the control. The most informative indicator of viability, the reproducibility of which did not depend on food conditions, was the percentage of reactivation, which was used in all further experiments. Long-term exposure (2–4 months) showed a gradual decrease in viability (the percentage of resting stage reactivation) (Figure 2). It is significant that a similar decrease was recorded in other species of aquatic invertebrates used in space experiments, in particular *Streptocephalus torvicornis* [29].

**Figure 3:**
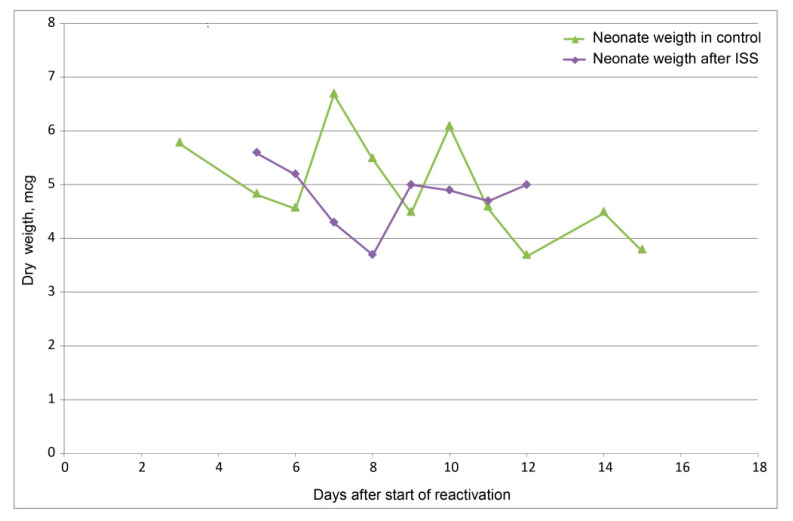
Dry weight of newborn daphnia after flight and in control. Dots illustrate the mean, *p* < 0.05.

A difference was also noted in the reactivation character and the value of the average weight of the individuals emerging from the resting eggs (Figure 2 and Figure 3). During reactivation after exposure in space, two rises were noted: the first one in the beginning was more significant, and the second one was on the 9th day, after some decline. At the same time, in both control series, only one maximum was noted, falling at the beginning of reactivation, after which there was a gradual decrease. The reactivation of the exposed individuals ended much earlier. In the control individuals, the reactivation began earlier due to the appearance of individuals smaller in dry weight. Among those exposed on the ISS, the first ascent was provided by larger embryos. During the period of reduced reactivation, the smallest individuals appeared, and the second slight rise was also provided by the individuals with the highest dry weight (see Figure 3).

In general, the number of the reactivated individuals in the experiment was significantly lower than in the control after a month’s exposure on the ISS: 39.6% in the ISS and 51.8% in the control, *t*-test, *p* = 0.035 [27]. As the duration of exposure in space increased, this difference also increased. Apparently, *D. magna* embryos on the ISS were exposed to a negative factor or a set of factors related to space flight, which suppressed their viability (a kind of stress caused by space flight); therefore, the weakest embryos with the lowest weight could not hatch first, and their place in the ISS group was taken by the large newborns, who may have also had a suppressed, but still sufficient, vital ability to hatch from their shells. This is confirmed by the experiments assessing the resistance of the control and experimental embryos to the parasitic fungus *Pythium daphnidarum* conducted by Petersen in 1910. The control individuals showed a noticeably greater resistance (in 1.43 times) to infection by the parasitic fungus than the individuals exposed to the ISS [27]. The significance of individual negative elements of the space flight factor that were encountered inside the spacecraft was investigated in the one-factor ground experiments discussed below.

The international program Phobos-Grunt, being predominantly geological (she had to bring a 1 m core from the surface of Phobos) [30], included a biological program to test for the survival of dormant stages of aquatic and terrestrial organisms, including *Daphnia magna*, for a long (about 2 years) exposure inside a spacecraft in open space conditions, unsoftened by the presence of Earth’s magnetic field. The returned part of Phobos-Grunt was supposed to deliver to Earth two sealed titanium containers that traveled to Mars’ orbit and back [31]. The container contained dry dormant stages of 50 species of organisms, including cyanobacteria, primitive fungi, diapausing eggs of daphnia, copepods, fairy shrimps, tadpole shrimps, ostracods, dipteran larvae, cysts of unicellular green algae and seeds of vascular plants. Unfortunately, for technical reasons, this interesting project was not implemented, and the Phobos-Grunt station, without breaking away from Earth’s orbit, ultimately sank in the Pacific Ocean. However, the orbital control of this interplanetary expedition became the Biorisk program [31,32], and all organisms that were part of the main expedition were exhibited on the outer surface of the ISS for 31 months. The results of this experiment confirmed the possibility of the long-term survival of dormant stages in open space [27]. The Russian space agency does not exclude the possibility of repeating the Phobos-Grunt program in the near future, including a biological sub-program on dormant stages.

## 3. Experiments Outside of the Spacecraft in Space

**Figure 4:**
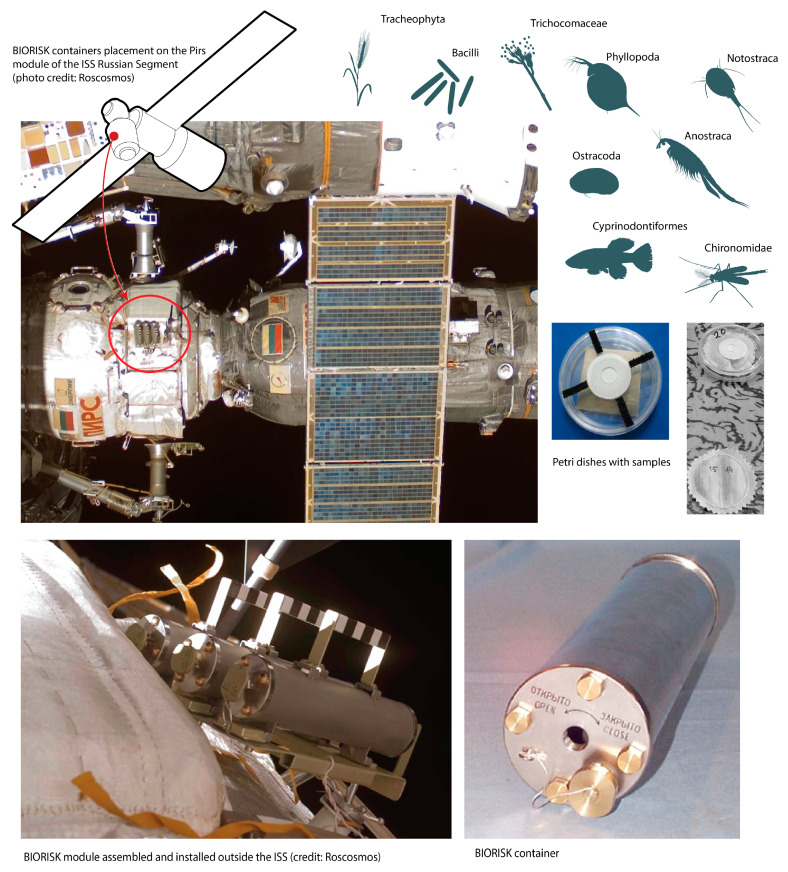
Biorisk program. Shows: experimental organisms; appearance of Biorisk containers; a Petri dish with biological samples before placing them in the container; location of the experimental module on the Pirs module of the ISS Russian Segment.

The Biorisk and Expose programs became, in fact, the predecessor of the Phobos-Grunt program for the survival of dormant stages of organisms in outer space. The Biorisk program was carried out in order to implement interplanetary quarantine to prevent the introduction of terrestrial life forms to other planets and back by unmanned and manned spacecraft [10,22]. The research kit is shown in Figure 4. Organisms, from bacteria to Cyprinodontiformes fish eggs, were placed in plastic containers with ventilation openings closed with bacterial membrane filters with pore sizes of 0.5 μm. Spores of bacteria and molds were applied to metal plates made of materials similar to those used to construct the shells of spacecraft. Plant seeds as well as the dormant stage of lower crustaceans and insects, were placed into cotton bags and then into plastic Petri dishes.

On 16 February 2007, the test objects were placed in three containers of Biorisk equipment, and the number and composition of the species in all containers were the same. The Biorisk device was attached to the outside of the Russian segment of the ISS by cosmonauts on 6 June 2007. The containers were to be returned to Earth at intervals of 6 months; however, due to a change in the plan for extravehicular activities, the first container was removed on 15 July 2008, i.e., in 13 months, not six as planned. It was returned to Earth on 24 October 2008 and was soon taken for research in laboratories in Moscow and St. Petersburg [33]. The second container was removed after 18 months of exposure, on 23 December 2008 and arrived at the ground laboratory on 9 April 2009. The third container was removed after 31 months on 14 January 2010.

The resting stages of brine shrimp showed the greatest survival in open space; however, the resting embryos of *Daphnia magna* were also able to survive the 13-month exposure under the conditions of multiple temperature changes from +100 to −100 °C in a vacuum and were exposed to the direct effect of electromagnetic and cosmic radiation as well as magnetic solar storms [10,22].

The Daphnia embryos that survived in such extreme conditions showed three types of damage, and only about 25% of the individuals were able to reproduce offspring capable of reproduction under Earth’s conditions.

The rest of the reactivated embryos in approximately equal proportions showed a violation of the life cycle (from the death of newborns to females reaching definitive sizes with an impossibility of laying eggs). The offspring obtained from those who successfully survived a long exposure in open space came from individuals with different dates of the onset of reactivation. They were studied by biochemical methods in order to identify intrapopulation polymorphism at protein loci [34]. It turned out that the individuals emerging from the eggs at the beginning and at the end of the reactivation period not only differed in body weight (the former were larger), but also belonged to different clones, obviously different in sensitivity to the stress factors of the space environment. Among those highly sensitive to cosmic stress, another group of clones with an increased level of the laying of resting eggs has emerged, under the same conditions as the rest, which can also be interpreted as an increased stress response, similar to the previously found increased male production upon the onboard exposure of eggs of this species. This result was confirmed in ground-based experiments with the effect of weak radiation on the production of *Daphnia magna* ephippia [35].

The resting stages of some species could not survive the long stay in outer space, which, in our opinion, was due to:

1. A high water content in some of the non-surviving species. This was indicated by the absence of reactivation in six variants of the non-dried *Chirocerpalus* sp. At the same time, among the dehydrated embryos of an ecologically closely related species, *Streptocephalus torvicornis*, reactivation ranged from 14 to 42% of the control;
2. Different depths of diapause. In species of the genus *Daphnia*, deep winter diapause is caused by a combination of the following factors: high population density, short day (12 h of light phase), poor nutritional conditions and the accumulation of bioinformation in the chain of generations through maternal transmission [36]. The clone of *Daphnia pulicaria* used in our experiment had weak superficial summer diapause induced by the only factor—deterioration of trophic conditions—and, as a result, all embryos died in the experimental groups. The reactivation of resting eggs in daphnia after their exposure in space was achieved only in embryos with deep (winter) diapause;
3. The tropical origin of species. Diapause of drying up Cyprinodontiformes fish eggs in nature lasts no more than 6–7 months to freezing. This apparently explains their complete mortality both in the experiment and control after 13 months. In a 6-month experiment, only fish embryos from the control group were reactivated and successfully developed. Since the experiment at the ISS planned for 6 months but lasted 13 months, and therefore was not delivered to Earth on time, it turned out to be impossible to separate the negative effects of the low temperatures and the long duration of exposure in space on the fish eggs’ survival [22].

Soon after the Biorisk program the European Space Agency (ESA) developed a more advanced multifunctional Expose device for testing the degradation of biological materials (amino acids) and spores of primitive organisms (bacteria and fungi) in space (inside meteorites) and under conditions on the surface of Mars [37]. Unlike the equipment of the Biorisk program, the Expose module was able to record or, in some cases, even regulate various environmental parameters: solar radiation, cosmic radiation and temperature [37,38].

**Figure 5:**
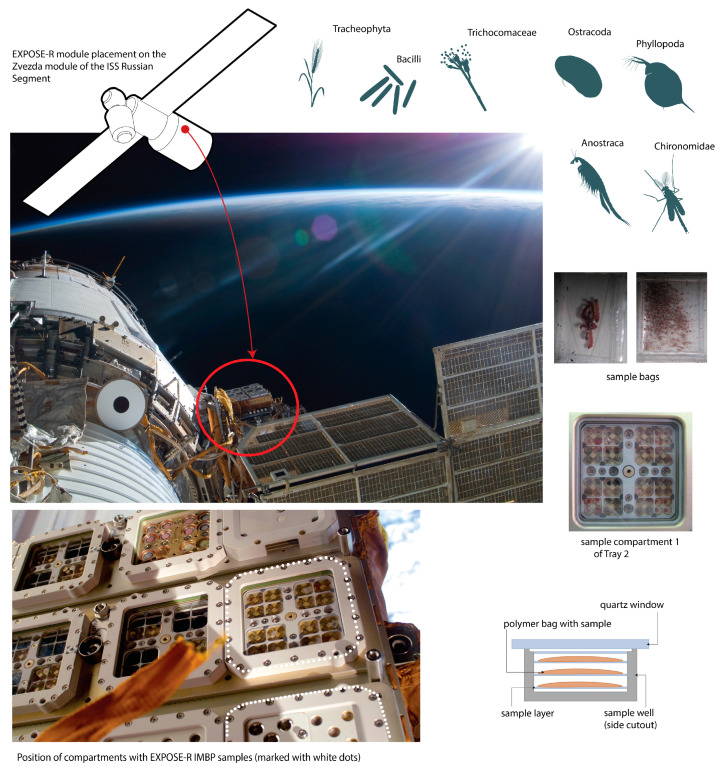
Expose-R program. Shows: experimental organisms; location of experimental module on the surface of the Zvezda module of the ISS Russian segment; module design; sealed plastic bags with biological samples.

The first module this series, Expose-E, was prepared for delivery to the ISS by the US National Aeronautics and Space Administration (NASA), launched on 7 February 2008, and returned to Earth on 12 September 2009. By this time, thanks to the Biorisk experiment, the high resistance of the resting stages of multicellular organisms to the conditions of open space had already become known. The next Expose-R project was developed by ESA in collaboration with Roscosmos and delivered to the ISS as a part of the Russian cargo in the Proton transport complex and placed outside the ISS on the Russian Zvezda module (Figure 5) [19].

The main differences between the investigated space conditions in the Expose program and Biorisk program are the softening of the temperature range and, at the same time, the possibility of assessing the impact of ultraviolet radiation of varying intensity.

Along with a set of biological objects prepared by ESA, Expose-R contained a Russian block with seeds of higher plants and dormant stages of several aquatic invertebrate species that survived in the Biorisk space experiments [19,20], including *Daphnia magna*.

All these objects were placed into small plastic bags in display wells in three layers separated from outer space by light filters of different permeability, which partly reduced the ultraviolet effect on the upper layer. In the Russian block of the experiment, the reduction in UV radiation by a quartz window was minimal [11,20].

After more than 1.5 years in outer space, the spores of microorganisms and fungi, seeds of two plant species (*Arabidopsis thaliana* and tomato *Lycopersicum esculentum*) and resting crustacean eggs (*Artemia franciscana*, *Eucypris ornata*, *Daphnia magna* and *Streptocephalus torvicornis*) were examined for viability and some parameters of the life cycle. Almost all of these organisms, both the aquatic and terrestrial species), located in the first layer of the samples in the Expose experiment, died, which was obviously due to exposure to solar radiation, since the background radiation was similar to Biorisk, and the temperature conditions controlled by the device were much milder. In the second layer, and especially in the third and last one, some of the organisms retained their viability [19,39]. Among the aquatic organisms, the greatest resistance, as in the experiments with Biorisk, was shown by the resting stages of *Artemia*, which is protected by several membranes and deep cryptobiosis.

The resting eggs of two crustacean species (*A. franciscana* and *E. ornata*) showed an increase in the percentage of surviving (reactivated) embryos with an increase in the distance from the first layer, which coincided with a weakening of ultraviolet radiation in this direction [27]. The resting eggs of two other species of aquatic crustaceans (*D. magna* and *S. torvicornis*) were much worse preserved even in the lower layers. A few of the surviving *D. magna* showed varying degrees of damage to the embryos, from death immediately after hatching to females attaining sexual maturity with an established inability to produce offspring.

Even the bacterial and fungal spores in the surface layer that were exposed to direct ultraviolet radiation died [37]. At the same time, in the third layer, where the exposure to the ultraviolet radiation was significantly reduced, the fungal spores showed very high survival rates (up to 100% compared with the control samples) [19]. Such a high survival was never found in the Biorisk experiments, where some species of fungi completely died, and the survival rate of the species that survived an 18-month flight did not exceed 0.5% [40]. In the Expose-R experiment, individual fungal spores were able to survive even in the upper layer in those species whose spores were more densely located and provided some protection from ultraviolet radiation, and, in the lower layers, survival was comparable to the control [19].

This shows, in our opinion, the role of the temperature factor and substrate in reducing the viability of the resting stages of these species, since in the Biorisk experiment the temperature fluctuated in the range from +100 to −100 °C with fungi spores being on a metal plate, and in Expose-R they were between +49 and −24 °C in polymer bags on quartz disks [19,40]. The softening of the temperature amplitude in the Expose-R experiment was almost threefold and did not go beyond the fluctuations of this factor observed under terrestrial conditions.

The experiment showed that not only bacterial and fungal spores, but also the dormant stages of multicellular organisms, such as plants and crustaceans, are able to withstand prolonged exposure to the factors of open space, including direct solar radiation, when provided minimal protection from UV radiation (e.g., cosmic dust or when in the shadow of the structural elements of vehicles or the depressions of meteorites of a complex shape).

To overcome the excessive negative (often fatal) effect of solar radiation on living organisms, the design of the Expose-R2 module was changed, with windows provided for the creation of various lower levels of ultraviolet irradiation of the required spectrum. The resting stages of two aquatic invertebrates (mosquito larvae *Polypedilum vanderplanki* and tadpole shrimp *Triops cancriformis*) were involved in Expose-R2, and no experiments were carried out with *Daphnia magna* ephippia. The results of these studies cannot be discussed as they have not yet been published. The Expose program ended in 2016. Theye are currently working on a new module called “Exobiology”, which should expand the possibilities of data collection “on the spot” [41].

## 4. Ground Experiments

The factor of space flight (SFF) inside the spacecraft, as it was accepted, consists of a number of environmental elements that negatively affect biological objects [42]. These include: microgravity, modified gas composition (for example, increased proportion of ethylene), cosmic and induced neutron radiation, modified magnetic field, radio and microwave radiation of working devices and mechanisms, mechanical noise and vibration, including ultrasound. The strength of the impact of the described SFF elements is quite different. Hereafter, we discuss the most significant of them, studied mainly using the example of the resting stages of *Daphnia magna.*

### 4.1. Microgravity

An important difference between the conditions of experiments in space, which is difficult to reproduce in terrestrial conditions, is the effect of microgravity, which always imposes a restriction on the study of the individual elements of the SFF in terrestrial laboratories. Nevertheless, aquatic organisms live on Earth also practically in zero gravity due to the high density of water. This also applies to the development of embryos of crustaceans and other organisms, which takes place in the fluid inside the egg. Even so, experiments with altered gravity did not reveal any noticeable deviations in the life cycle of the studied organisms [43]. These results, as well as an analysis of the living conditions of organisms in the aquatic environment, allowed us to conduct laboratory studies of other elements of the SFF in Earth’s conditions and to take into account the minimal effect of microgravity on the resting stages of aquatic organisms in space flight. This conclusion should not be opposed to the results obtained for terrestrial organisms, in which the absence of weight turns out to be one of the most important elements of the factor of space flight, causing a weakening of the musculoskeletal system in animals, redistribution of moisture in the tissues of living organisms and changing the formation of the root system in higher plants [44].

### 4.2. Gas Composition Inside of Vehicle

The gas composition inside the ship can also be excluded as insignificant due to the impermeability of the sample package and the almost complete absence of metabolism in most dormant stages, including *Daphnia magna* [45].

### 4.3. Cosmic Radiation

It is rather difficult to recreate cosmic (corpuscular) radiation in ground-based experiments. This requires the acceleration of elementary particles in synchrophasotrons to speeds close to the speed of light. Nonetheless, the density of such particles in space is relatively low, and, while passing through the spaceship, they collide with the molecules of gases and objects located there, causing the appearance of a certain amount of neutrons (secondary radiation), which mainly affects biological objects [46].

**Figure 6:**
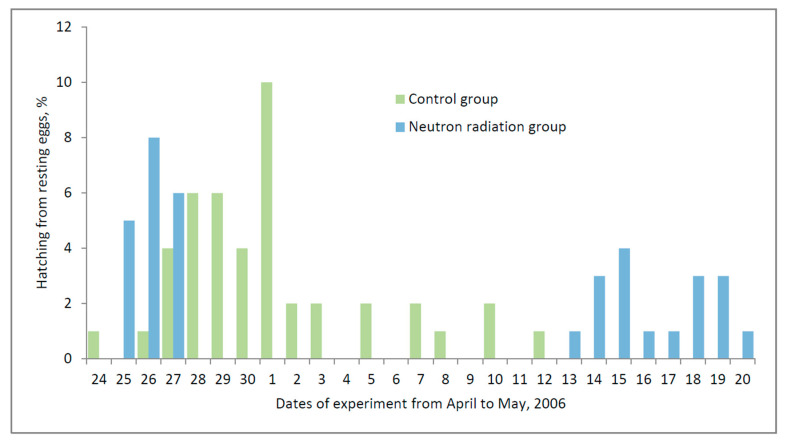
Effect of neutron radiation on hatching of dormant stages of *D. magna*. Bars illustrate the mean, *p* < 0.05.

To recreate a radiation environment similar to that on board the ISS in ground-based experiments, a neutron radiation source of equivalent power (about 200 μGy) was used, which is approximately twice the maximum value of the natural background radiation on the Earth’s surface. The resting stages of *Daphnia magna* were exposed under these conditions for two weeks [1,28]. The embryo reactivation lasted for a month and was divided into two stages (Figure 6). During the first stage, the reactivation of the neutron-treated ephippia was close to that of the ISS group (the first two weeks), but it was significantly lower than in the control. After 2 weeks, the reactivation of the second group of ephippia began, which had postponed hatching. The summarizing of the results of the first and second groups, in terms of the total number of reactivated individuals, did not differ statistically from the control. In the experiments on the ISS, two periods of the release of *Daphnia magna* from the embryos were also noted: at the beginning of reactivation and some time after it. These were separated by a period of noticeable decline, but this period of decline was significantly shorter than the interval between the first and second peaks in the ground experiments. The control samples did not show such a decrease either in the space experiment or in the ground one.

It seems that exposure to radiation as a stress signal caused a noticeable delay in the reactivation in one part of the *Daphnia* population. Subsequent studies of these two parts of the *Daphnia* population showed that they belong to different clones, as mentioned above [34].

Despite a clear delay in the reactivation of ephippia *D. magna* by the second clone, the total number of survivors after this exposure did not differ from the control, and the negative effect on the reactivation/viability should be assessed as low. Based on these results, the negative impact of cosmic radiation of such intensity has been exaggerated, and damage to biological objects during a long flight, for example to Mars and back, may not be critical for their health. Nevertheless, the cumulative and, possibly, synergistic (with other weak elements of SFF) impacts of space radiation on biological objects require further study.

### 4.4. Modified Magnetic Field

To assess the impact of a modified Earth’s magnetic field during space flight, in the experimental setup of the Pushkov Institute of Terrestrial Magnetism, Ionosphere and Radiowave Propagation of the Russian Academy of Sciences (IZMIRAN), the resting stages of *D.* *magna* were exposed for 15 days in three experimental variants and in control: 1—no magnetic field; 2—a doubled Earth’s magnetic field; 3—a modified electromagnetic field simulating the operation of electrical devices of the ISS. The resting stages of *D. magna* in the natural magnetic field ofEarth served as a control. The experiments were carried out in June 2008, at an exposure temperature of 20–22 °C with the natural light conditions of a given latitude of the area (58 N) at this time (14–28 June, maximum day length) [27]. Each of the variants was performed in triplicate, including at least 70 *D. magna* embryos in each, selected from the same sample that was used in the experiments on the ISS. After the end of the exposure, the resting *D. magna* eggs were reactivated and compared with the control. A statistical analysis did not reveal differences in the rate of reactivation and the proportion of genetically modified individuals among of all these variants and the control. A more detailed analysis of the effect of the magnetic field on other elements of the life cycle on another clone of *D. magna* was carried out by Krylov [47]. He investigated the most important parameters of the life cycle of *D. magna* concerning daphnia growth and reproduction in a modified magnetic field of Earth or electromagnetic fields of variated frequency and power, namely: attainable definitive sizes of females, number of eggs produced (clutch size), size of newborn juveniles and age of the female at the time of formation of the first clutch. There were also no statistically significant differences in any of these parameters in all of the magnetic field variants studied.

In experiments with other organisms (roach), where, along with a modified magnetic field, the light conditions also changed, some significant differences were found: an increase in the length of the larva after hatching and some teratogenic changes during mitosis. These changes occurred primarily when the light conditions were modulated; however, when the factor of the modified magnetic field was added to them, the effect increased. At the same time, a modified magnetic field alone did not have a noticeable effect on these parameters [48].

This makes it possible to classify the modified magnetic field as a very weak effect, which, nevertheless, can lead to synergistic influence and modify the effect of other stronger factors.

### 4.5. Ultrasound and Microwave

The noise (vibration), radio waves and high-frequency radiation associated with the operation of the mechanisms and electrical devices of the ISS should also be attributed to the possible factors, the perception of which is not impeded onboard the spacecraft by the isolation of the resting stages of organisms in plastic bags.

From the above list, the effect on *D. magna* reactivation was studied in two of the most significant elements of the SFF: microwave and ultrasound. Within the limits of the parameters for these indicators, allowable by most federal safety standards (1–5 mW/cm^2^), no significant effect from both of the parameters acting separately or together on the reactivation of *D. magna* was found [27].

Other researchers evaluating the effect of non-ionizing radiation exposure with a low intensity found some contradictory results associated with fluctuations in the daphnia’s fecundity (an increase and then decrease) and change in sensitivity to toxicants (it could increase or decrease depending on the active toxic agent) [49,50].

In general, even a prolonged (up to 45 min) exposure to weak microwave fields that do not go beyond the values permissible, in terms of sanitary and hygienic conditions, should be attributed to the absent or weak effects with an assumed ability for synergy in the formation of the general factor of space flight. However, it is difficult to quantify the impact or even its direction at the moment. Nevertheless, going beyond the permissible values can be harmful to the biological objects inside the ship.

## 5. Conclusions

Studies of the individual elements of the space flight factor that manifested themselves during intra-cabin transportation of the resting stages did not reveal a significant effect of each of the elements, considered separately, on the reactivation of embryos.

The most significant was the effect of neutron radiation, which during the experiments did not affect the survival of the embryos, but undoubtedly was accepted by them, which led to a strong extension of the total reactivation period and a difference in the rate of hatching comparing with the control group. It should be noted that the ground-based experiment with exposure to neutron radiation lasted 14 days only, while the shortest experiment in orbit, which revealed significant differences between the exposed and control groups (14% ± 3.2), lasted a month. After 8 months of exposure in space, the difference between the experimental and control groups increased, and the decrease in the percentage of reactivation in different species was from 30 to 70% (on average 50% ± 13.8) compared with control group.

Considering that low doses of radiation on biological objects has a cumulative effect, it can be assumed that a monthly stay in orbit (twice as long as the ground experiment) already led to a significant difference in the degree of survival of the resting embryos and became the basis for the further decrease in the survival rate of organisms.

Another possible explanation for the growing difference in the survival of the dormant stages with prolonged exposure on the ISS may be the synergistic effect of the action of the other weak elements of the SFF, which, when acting together, increases the effect of the most powerful one. This possibility was established when studying the combined effect of the changed magnetic field and light conditions on the viability of roach larvae (*Rutilus rutilus*) [48]. In general, the synergistic effect of the weak elements of the SFF undoubtedly requires further study.

An important consequence of the experiments inside the ISS was the proven possibility of delivering the dormant stages of an artificial ecosystem to other planets in a viable state, which will take up to 8 months or even longer. Despite a noticeable increase in the mortality percentage, the organisms obtained from the surviving embryos had the ability to successfully reproduce and increase the population size.

The experiments on the outer surface of the ISS revealed that the most powerful factor in reducing the survival of the resting stages of various organisms was solar radiation, which was capable of destroying up to 100% of the spores of bacteria and fungi, as well as the most resistant multicellular organisms. However, with minimal protection from direct exposure to ultraviolet radiation, the chance for survival of these organisms in open space increases significantly even in the conditions of a vacuum and a two-hundred-degree temperature range. This increases the need to follow interplanetary quarantine when visiting other planets, both in inhabited and unmanned vehicles.

Alternatively, these results allow us to make some predictions about the search for possible extraterrestrial life on other planets and asteroids.

The highest chances of survival on celestial bodies (Mars, Mercury and asteroids) actively irradiated by the Sun will be in places protected from direct exposure to ultraviolet radiation—areas with permanent shade, caves, depressions in the surface layer and subsurface ecosystems. A necessary condition for such specific ecosystems is also at least a periodic possibility of the existence of water in the liquid phase to maintain the active period of the development of such organisms.

## Figures and Tables

**Table 1:** List of organisms that participated in Russian experiments on the ISS and conditions of dormancy induction.

Taxon	Akvarium	Biorisk	Expose-R	Conditions of Dormancy Induction
Animalia, Arthropoda	*Daphnia magna*	*Daphnia magna*	*Daphnia magna*	Natural conditions during the end of spring: starvation and high temperature (up to 40 °C)
		*Daphnia pulicaria*		Aquarium conditions: short day length, starvation
	*Streptocephalus torvicornis*	*Streptocephalus torvicornis*	*Streptocephalus torvicornis*	Same as in *D. magna*
		*Artemia salina*	*Artemia salina*	High temperature, increasing salinity from 50 to 200‰
		*Eucypris ornata*	*Eucypris ornata*	Same as in *D. magna*
		*Triops cancriformis*		Stock culture, conditions of dormancy unknown
		*Chirocephalus* sp.		Natural conditions: drying up during summer time
		*Polypedilum vanderplanki*	*Polypedilum vanderplanki*	Aquarium conditions: drying up in summertime
Animalia, Chordata		*Nothobranchius guentheri*		Aquarium conditions: photoperiod 14:10, permanent temperature 25 °C
Bacteria, Firmicutes		*Bacillus subtilis*, *B. licheniformis*	*Bacillus subtilis*, *B. licheniformis*, *B. pumilus*	Drying up at room temperature
Fungi, Ascomycota		*Penicillium expansum*, *P. aurantiogresium*, *Aspergillus versicolor*, *A. sedowii*	*Penicillium expansum*, *P. aurantiogresium*, *Aspergillus versicolor*, *A. sedowii*	Drying up at room temperature
Plantae, Tracheophyta		*Brassica rapa*, *B. juncea*, *Arabidopsis thaliana*, *Nicandra physalodes*, *Lycopersicum esculentum*, *Raphanus sativus*, *Hordeum vulgare*, *Oryza sativa*	*Arabidopsis thaliana*, *Lycopersicum esculentum*	Drying up at room temperature

**Table 2:** Environmental conditions of experiments with dormant stages on the ISS.

	On Board ISS	Outside ISS	
Program	Akvarium	Biorisk	Expose-R (RusPart)
Exposure duration, days	30, 240	405–935	682
Temperature, °C	+17 … +28 [9]	−100 … +100 [9,10]	−24 … +49 [11]
Gravity	~1 μg (microgravity)		
Magnetic field, μT	~40 [12]		
Exposure to UV-B and UV-C radiation	absent, sample holder is lightproof	absent, sample holder is lightproof	present, 2687 h (100% transmission λ > 170 nm) [11,13]
Average dose of cosmic radiation per day, μGy/day	180–360 [14]	320–408 [15,16]	323–381 [17,18]

## Data Availability

The data that support the findings of this study are available from the authors upon reasonable request.
